# A New Cycloartane-Type Triterpenoid Saponin Xanthine Oxidase Inhibitor from *Homonoia riparia* Lour 

**DOI:** 10.3390/molecules190913422

**Published:** 2014-08-29

**Authors:** Fan Xu, Xueqian Zhao, Lingli Yang, Xiuhua Wang, Jing Zhao

**Affiliations:** 1 Kunming General Hospital of Chengdu Military Region, Kunming 650032, Yunnan, China; E-Mails: xu_fan@126.com (F.X.); zhaoxueqian@126.com (X.Z.); wangxh2341@126.com (X.W.); 2 The First Affiliated Hospital of Kunming Medical University, Kunming 650031, Yunnan, China; E-Mail: lingli_yang@126.com

**Keywords:** *Homonoia riparia* Lour, cycloartane-type triterpenoid saponin, xanthine oxidase inhibitors

## Abstract

A new cycloartane-type triterpenoid saponin named riparsaponin (**1**) was isolated from the stem of *Homonoia riparia* Lour together with six known compounds. The structure of riparsaponin was determined by using NMR and mass spectroscopy and X-ray crystallography techniques. Additionally, riparsaponin has a significant inhibitory effect on xanthine oxidase *in vitro*, and the IC_50_ was 11.16 nmol/mL.

## 1. Introduction

**Figure 1:**
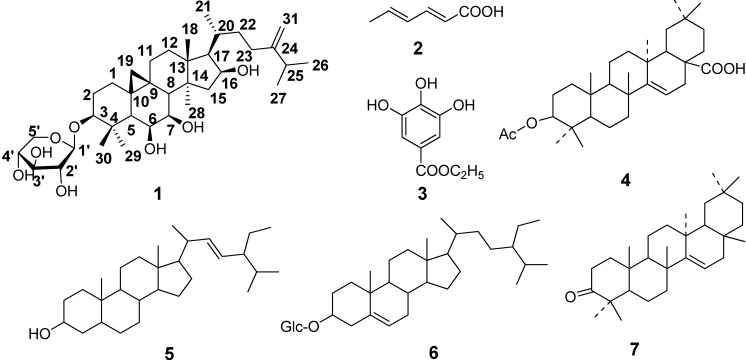
Structure of the compounds isolated from *H. riparia*.

*Homonoia riparia* Lour, (family Euphorbiaceae), is widely distributed in the south part of China [1]. The roots of *H. riparia* are commonly used as an effective traditional Chinese herbal medicine for treating hepatitis and joint gall, stomach ache, and ambustion based on its antipyretic choleretic, anti-inflammatory, detoxification, and diuretic activities [1,2]. However, phytochemical and pharmacological investigations of this plant are currently lacking. Previous chemical research reported that it contains triterpenes, steroids, and phenolics [3,4,5]. In our present investigation, a new cycloartane-type triterpenoid saponin (compound **1**, named riparsaponin) was isolated from the stems of *H. riparia* together with six known compounds (Figure 1). In addition, riparsaponin showed significant inhibitory activity on xanthine oxidase. Here, we report the isolation, identification and activity of the new compound, which could be helpful for treating gouty arthritis.

## 2. Results and Discussion

### 2.1. Identification of the Riparsaponin

**Figure 2:**
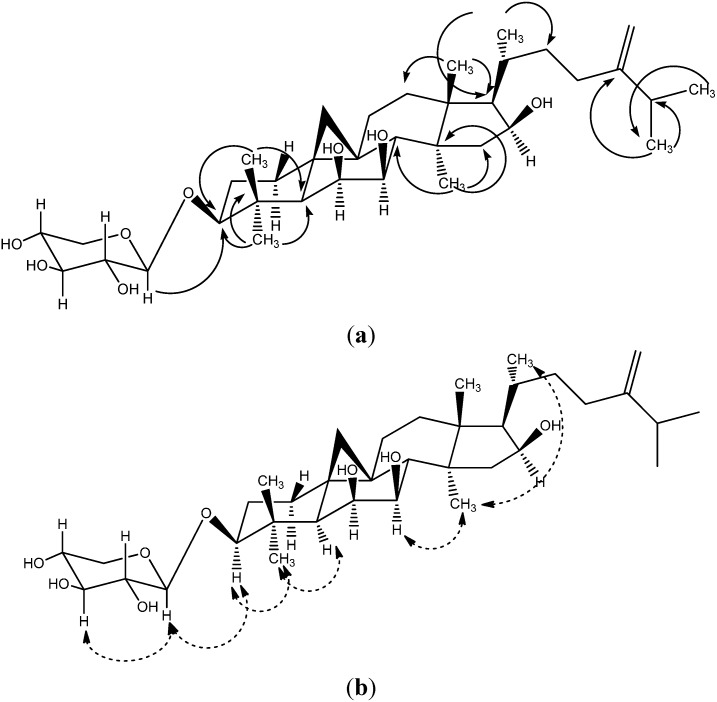
The key HMBC (**a**) and ROESY (**b**) connections of riparsaponin (**1**).

**Figure 3:**
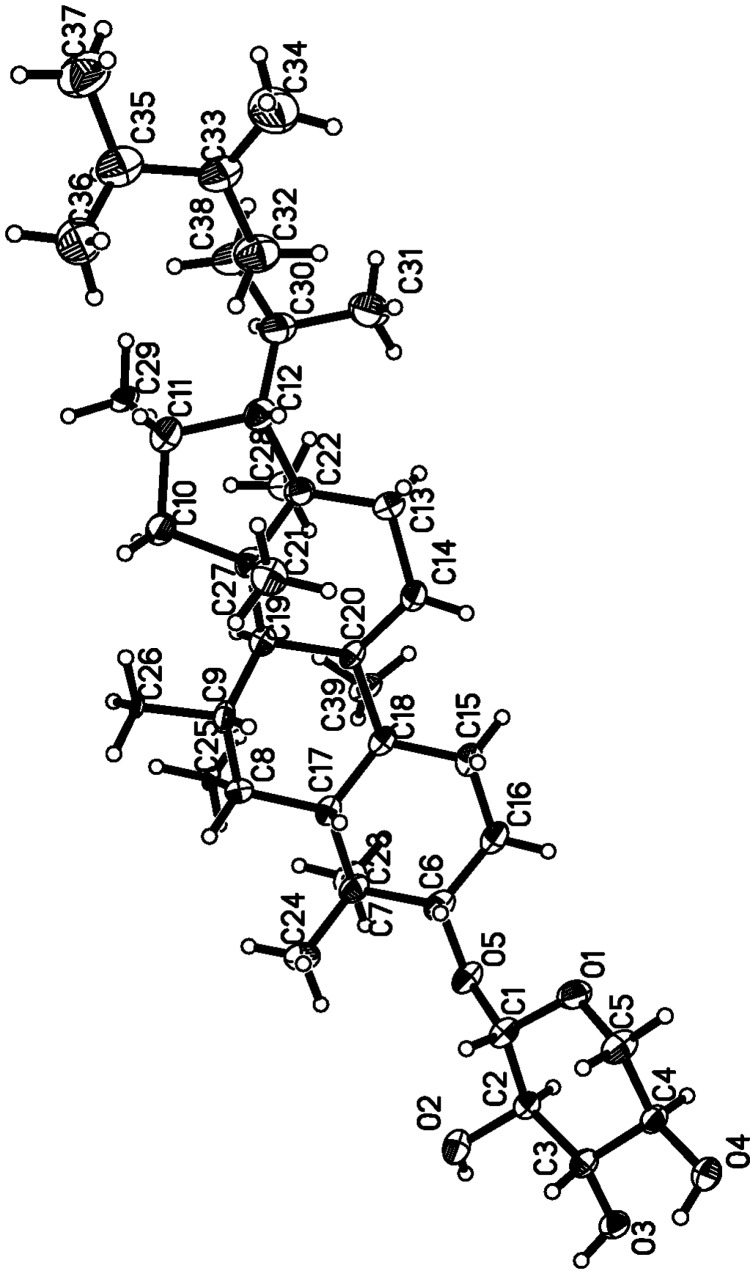
Perspective drawing of compound **1** generated from X-ray crystal data.

The new compound riparsaponin (**1**) was identified by using NMR and mass spectroscopy, and X-ray crystallography techniques. Compound **1** was obtained as colorless prismatic crystal (MeOH); mp: 291–292 °C. Its Liebermann-Burchard response was positive. FAB-MS (*m/z*) (%): 620 (99), 712 (29), 487 (55). The ^13^C-NMR, ^1^H-NMR data are listed in Table 1. The ^13^C-NMR spectrum (100 MHz, DMSO-*d*_6_) showed 36 carbon signals, and lots of carbon signals were between 60–80 (δ_C_), which indicated the existence of a saccharide group. The carbon signals of 105.9, 73.82, 105.9, 73.82, and 65.62 (δ_C_) could further demonstrate the existence of pentose, and the carbon signal at 105.9 ppm is the terminal group carbon of the pentose (β-type) [6]. The two high-field doublets observed at δ_H_ 0.99 (1H, m) and 0.3 (1H, s) are characteristic of the two germinal protons of a cyclopropane moiety. What’s more, two olefinic protons at δ_H_ 4.65 (2H, brs) indicated the presence of an olefinic methylene moiety. There are two carbon signals at 106.13 and 156.22 (δ_C_), suggesting a double bond. The ^1^H-NMR spectrum (400 MHZ, DMSO-*d*_6_) indicated seven methyl groups (δ_H_ 0.87, 0.88, 0.96, 0.98, 1.05, 1.08, 1.12). These spectroscopic data suggested this compound to be a cycloartane-type triterpene with an exocyclic methylene group. Combined the reference data [3], we found the ^1^H-NMR and ^13^C-NMR spectra of compound **1** were analogous to that reported for a cycloartane-type triterpene which was isolated from *H. riparia* previously. Furthermore, the structure of compound **1** was confirmed by HMBC, ROESY (Figure 2) and X-ray diffraction (CCDC deposition number is **1021164**) (Figure 3, Table 2).

**Table 1:** ^1^H-NMR (400 Hz) and ^13^C-NMR (100 Hz) data of riparsaponin in DMSO-*d*_6_.

	δ_C_	δ_H_		δ_C_	δ_H_
**1**	32.62 t	1.53 (2H, m)	19	31.41 t	0.99 (1H, m)
					0.30 (1H, s)
**2**	29.16 t	1.72 (1H, m)	**20**	29.77 d	1.77 (1H, m)
		1.53 (1H, m)			
**3**	87.52 d	3.00 (1H, m)	**21**	17.92 q	0.87 (3H, d, *J* = 8.6 Hz)
**4**	40.82 s		**22**	34.54 t	1.80 (1H, m)
					1.08 (1H, m)
**5**	49.02 d	1.22 (1H, s)	**23**	31.35 t	2.07 (1H, m)
					1.88 (1H, m)
**6**	71.38 d	3.74 (1H, brs)	**24**	156.22 s	
**7**	72.74 d	3.21 (1H, m)	**25**	33.15 d	2.22 (1H, m)
**8**	45.90 d	1.88 (1H, d, *J* = 11.2 Hz)	**26**	21.76 q	0.96 (3H, s)
**9**	24.04 s		**27**	21.81 q	0.98 (3H, s)
**10**	18.72 s		**28**	19.43 q	0.88 (3H, s)
**11**	25.43 t	1.91 (1H, m)	**29**	24.07 q	1.05 (3H, s)
		0.92 (1H, m)			
**12**	32.27 t	1.36 (1H, m)	**30**	16.40 q	1.08 (3H, s)
		1.08 (1H, m)			
**13**	45.64 s		**31**	106.13 t	4.65 (2H, brs)
**14**	45.41 s		**1**'	105.90 d	4.09 (1H, d, *J* = 7.5 Hz)
**15**	51.66 t	2.16 (1H, m)	**2**'	73.82 d	2.95 (1H, m)
		1.41 (1H, m)			
**16**	70.72 d	4.14 (1H, m)	**3**'	76.79 d	3.04 (1H, m)
**17**	55.44 d	1.45 (1H, m)	**4**'	69.66 d	3.24 (1H, m)
**18**	19.39 q	1.12 (3H, s)	**5**'	65.62 t	3.61(1H, m)
					2.97 (1H, m)

**Table 2:** Crystal data and structure refinement for riparsaponin.

Identification code	070516a
Empirical formula	C_36_H_57_O_8_
Formula weight	617.82
Temperature	298 (2) K
Wave length	0.71073 A
Crystal system, space group	Orthorhombic, P2(1)2(1)2(1)
Unit cell dimensions	a = 6.3405(9)Aalpha = 90 deg.
	b = 12.7265(17)Abeta = 90 deg.
	c = 41.573(6)Agamma = 90 deg.
Volume	3354.6(8) A^3^
Z, Calculated density	4, 1.223 Mg/m^3^
Absorption coefficient	0.085 mm^−1^
F (000)	1348
Crystalsize	0.26 × 0.22 × 0.08 mm
The tarange for data collection	1.67 to 28.31 deg.
Limiting indices	−8 ≤ h ≤ 8,−16 ≤ k ≤ 16,−55 ≤ l ≤ 53
Reflections collected/unique	29112/8026[R(int) = 0.0893]
Completeness to theta = 28.31	98.9%
Absorption correction	MUTI-SCAN
Max. andmin. transmission	1.000000 and 0.832723
Refinement method	Full-matrixleast-squaresonF^2^
Data/restraints/parameters	8026/0/398
Goodness-of-fitonF^2^	0.786
Final Rindices [I > 2sigma(I)]	R_1_ = 0.0666, wR_2_ = 0.1877
Rindices (alldata)	R_1_ = 0.1410, wR_2_ = 0.2517
Absolute structure parameter	0.7(17)
Extinction coefficient	0.0040(15)
Largest diff. Peak and hole	0.342 and −0.329 eA^−3^

### 2.2. Inhibitory Effect of Riparsaponin on Xanthine Oxidase Activity in Vitro

Gout is one of the common human metabolic diseases and caused by hyperuricemia, which can result in depositions of urate crystals in joints, leading to gouty arthritis [7]. Xanthine oxidase plays an important role during the formation of uric acid, and the accumulation of uric acid can result in hyperuricaemia, leading to gout [8]. Previous investigations revealed that inhibitors of xanthine oxidase could be potentially beneficial for treating gouty arthritis [9]. Herbal remedies have been used in China for more than millennium, and lots of investigations have reported that the herbal medicines and its derived compounds can safely and effectively in treatment of various diseases [10,11]. In our present study, the inhibitory activities of the six known compounds on xanthine oxidase were weak, but riparsaponin could significantly inhibit xanthine oxidase activity *in vitro* at the doses during 9.68 to 161.29 nmol/mL compared with the DMSO group (*p* < 0.01), in a dose-dependent manner (Table 3). In our present study, the IC_50_ of riparsaponin was 11.16 nmol/mL, which is a better value compared to allopurinol used as positive control drug (IC_50_ 11.84 nmol/mL). The results above indicated that riparsaponin is a potential powerful xanthine oxidase inhibitor.

**Table 3:** Inhibitory effect of riparsaponin on xanthine oxidase activity (n = 5).

Group	Concentration (nmol/mL)	Fluorescence Unit	Inhibition Ratio (%)	IC_50_ Value (nmol/mL)
DMSO		8.291 ± 0.892	0.07	
Riparsaponin	4.84	7.205 ± 0.865	13.09	11.16
	9.68	4.815 ± 0.861 **	41.93	
	19.36	3.248 ± 0.912 **	60.82	
	40.32	2.362 ± 0.127 **	71.52	
	80.65	1.872 ± 0.483 **	77.42	
	161.29	1.474 ± 0.131 **	82.22	
Allopurinol	2.20	6.796 ± 0.696 *	18.03	11.84
	4.41	6.212 ± 0.580 **	25.08	
	8.82	4.879 ± 0.410 **	40.93	
	17.63	2.776 ± 0.217 **	66.52	
	35.27	2.062 ± 0.370 **	75.13	
	70.53	1.617 ± 0.220 **	80.49	

Data were expressed as Mean ± SD, * *p* < 0.05, ** *p* < 0.05, compared to the DMSO group.

## 3. Experimental Section

### 3.1. General Information

These following instruments were used: UV visible spectrophotometer (UV-1600) was made by Rayleigh Analytical Instrument Company (Beijing, China). Mass spectrometer (EI-MS) (VGAutoSpec-3000, Beckman, CA, USA). The NMR (AV-400, AV-500) and X-ray single crystal diffractometer (APEX II DUO) instruments were both made by Bruker (Bremen, Germany).

### 3.2. Plant Material

The stems of *H. riparia* were collected in Jinping County, Yunnan Province, China, in October 2010. The plant was identified by Jingxiu Li (Kunming Institute of Botany Chinese Academy of Science, Kunming, Yunnan, China). A voucher specimen was deposited in our laboratory.

### 3.3. Extraction and Isolation

Dried stems of *H. riparia* (58.8 kg) were powered and extracted three times with 60% ethanol (total 300 L) at reflux. Then the extracts were spray-dried to obtain a dry fine powder (4.8 kg). The powder was extracted three times by maceration with petroleum ether, CH_2_Cl_2_, EtOAc, and 95% ethanol, respectively (total 40 L, each extraction lasted 2 days).

After concentration, compound **2** was crystallized from the petroleum ether fraction, and the pure compound **2** (3.17 g) was obtained by recrystallization from EtOAc. Then, the petroleum ether fraction (73 g) was subjected to column chromatography (CC) over silica gel (200–300 mesh) eluting with petroleum ether–EtOAc (9:1, 8:1, 5:1, 3:1, 1:1, 1:3, 1:5, 1:9), and seven sub-fractions **A**–**G** were obtained on the basis of TLC analysis. Compound **4** was crystallized from fraction **B**, and the pure compound **4** (60 mg) was obtained by recrystallization from petroleum ether–CH_2_Cl_2_ (9:1). Fraction **C** was subjected to CC over silica gel (200–300 mesh) eluting with petroleum ether–CH_2_Cl_2_ (9:1, 8:1, 5:1, 3:1, 1:1, 1:3, 1:5, 1:9), and combination of similar fractions after TLC comparison afforded five fractions **C_1_**–**C_5_**. Compound **5** was crystallized from fraction **C_2_**, and the pure compound **5** (87 mg) was obtained by recrystallization from EtOAc.

The CH_2_Cl_2_ fraction (100 g) was subjected to AB-8 macroporous resin (The Chemical Plant of Nankai University, Tianjing, China) CC eluting with gradient ethanol (0%, 30%, 70%, 90%), and four fractions **A**–**D** were obtained. After concentration, compound **3** was crystallized from fraction **D**, and the pure compound **3** (45 mg) was obtained by recrystallization in petroleum ether–EtOAC (9:1). Fraction **C** was subjected to CC over silica gel (200–300 mesh) eluting with petroleum ether–acetone (9:1, 8:1, 5:1, 3:1, 1:1, 1:3), and four sub-fractions **C_1_**, **C_2_**, **C_3_**, **C_4_** were obtained on the basis of TLC. Sub-fraction **C_2_** was subjected to CC over silica gel (200–300 mesh) eluting with petroleum ether–acetone (9:1, 8:1, 5:1, 3:1, 1:1, 1:3) again to afford four sub-fractions **C_21_**–**C_24_**, and compound **6** (white powder, 144 mg) was separated out from fraction **C_22_**.

The EtOAc fraction (72.3 g) was subjected to CC over silica gel (200–300 mesh) eluting with petroleum ether–acetone (15:1, 10:1, 7:1, 5:1, 3:1, 1:1, 1:2), to give six sub-fractions **A**–**F** on the basis of TLC analysis. After concentration, compound **7** (a white powder, 24 mg ) was separated out from fraction **E**. Fraction **B** was repeatedly subjected to CC over silica gel (200–300 mesh) eluting with petroleum ether–acetone (10:1, 7:1, 5:1, 3:1, 1:1, 1:2), and four sub-fractions **B_1_**–**B_4_** of **B** were thus obtained. Then sub-fraction **B_2_** was subjected to CC over silica gel again eluting with a gradient of petroleum ether–acetone, and five fractions **B_21_**–**B_25_** were obtained; then compound **1** was crystallized from **B_22_**, and the pure compound **1** (213 mg) was obtained by recrystallization from petroleum ether–EtOAC.

*2,4-Sorbic acid* (**2**) was obtained as colorless crystals (EtOAc), mp: 134–136 °C; EI-MS (*m/z*) (%): 111 (38), 97 (71), 83 (80), 69 (90), 57 (100); ^1^H-NMR (400 MHz, CDCl_3_, ppm) δ: 5.75 (1H, d, *J* = 15.3 Hz, H-2), 7.33 (1H, dd, *J* = 15.4, 9.7 Hz, H-3), 6.26 (1H, m, H-4), 5.79 (1H, s, H-5), 1.87 (3H, d, *J* = 5.0 Hz, CH_3_-6); ^13^C-NMR (100 MHz, CDCL_3_, ppm) δ: 173.5 (C-1), 118.4 (C-2), 147.7 (C-3), 130.0 (C-4), 141.2 (C-5), 19.1 (C-6).

*Ethyl gallate* (**3**) was obtained as colorless crystals [petroleum ether‒EtOAc (9:1)], mp: 169–170 °C; FAB-MS (*m/z*) (%): 197 (90), 169 (5), 154 (100), 124 (3), 97 (3), 80 (15), 64 (3); ^1^H-NMR (400 MHz, CD_3_ODCD_3_, ppm) δ: 7.11 (2H, s, H-2, H-6), 4.26 (2H, q, *J* = 7.0 Hz, OCH_2_CH_3_), 1.27 (3H, t, *J* = 7.2 Hz, OCH_2_CH_3_), 6.94 (2H, d, *J* = 1.0 Hz), 8.12 (1H, s), 9.25 (2H, s); ^13^C-NMR (100 MHz, CD_3_ODCD_3_, ppm) δ: 122.0 (C-1), 109.7 (C-2), 146.0 (C-3), 138.6 (C-4), 146.0 (C-5), 109.7 (C-6), 166.6 (C-7), 60.9 (C-8), 14.6 (C-9).

*Acetylaleuritolic acid* (**4**) was obtained as colorless crystals [petroleum ether‒CH_2_Cl_2_ (9:1)], mp: 287–288 °C; EI-MS (*m/z*) (%): 498 (M^+^, 3), 439 (M^+^-COCH_3_, 2), 344 (RDA fragment, 5), 285 (344-COCH_3_), 189(100). ^1^H-NMR (400 MHz, CDCl_3_, ppm) δ: 0.75 (3H,s, CH_3_-26), 0.84 (3H, s, CH_3_-23), 0.88 (3H, s, CH_3_-24), 0.91 (3H, s, CH_3_-29), 0.93 (3H, s, CH_3_-30), 0.95 (3H, s, CH_3_-25), 1.13 (3H, s, CH_3_-27), 2.04 (3H, -COCH_3_), 4.48 (1H, t, *J* = 8.0 Hz, H-3), 5.50 (1H, t, *J* = 3.3 Hz, H-15); ^13^C-NMR (100 MHz, CDCl_3_, ppm) δ: 37.9 (C-1), 22.4 (C-2), 80.9 (C-3), 37.7 (C-4), 55.3 (C-5), 18.1 (C-6), 33.6 (C-7), 39.0 (C-8), 47.5 (C-9), 37.0 (C-10), 23.4 (C-11), 32.4 (C-12), 41.3 (C-13), 160.5 (C-14), 116.8 (C-15), 23.5 (C-16), 46.5 (C-17), 41.3 (C-18), 45.8 (C-19), 30.6 (C-20), 32.4 (C-21), 27.9 (C-22), 28.6 (C-23), 16.6 (C-24), 15.6 (C-25), 17.3 (C-26), 25.9 (C-27), 184.2 (C-28), 33.0 (C-29), 23.6 (C-30), 171.0 (-COOH), 21.3 (-COCH_3_) [12].

*Spinasterol* (**5**) was obtained as colorless crystals (CH_2_Cl_2_), mp: 162–163 °C; EI-MS (*m/z*) (%): 412 (M^+^), 396 (M^+^-O), 351 (369-OH), 271 (300-C_2_H_5_), 255 (271-O), 213 (255-C_3_H_6_); ^1^H-NMR (400 MHz, CDCl_3_, ppm) δ: 5.15 (1H, H-7), 5.03 (1H, dd, *J* = 8.6 Hz, H-22), 5.21 (1H, dd, *J* = 8.5 Hz, H-23), 0.70 (3H, s, CH_3_-18), 0.81 (3H, s, CH_3_-19), 0.83 (3H, t, *J* = 7.3 Hz, CH_3_-29), 0.85 (3H, d, *J* = 6.3 Hz, CH_3_-26), 0.86 (3H, d, *J* = 6.3 Hz, CH_3_-26), 1.04 (3H, d, *J* = 6.6 Hz, CH_3_-21), 3.54 (H, tt, *J* = 11.0 Hz, H-3); ^13^C-NMR (100 MHz, CDCl_3_, ppm) δ: 37.2 (C-1), 31.6 (C-2), 71.8 (C-3), 37.9 (C-4), 40.5 (C-5), 29.6 (C-6), 121.7 (C-7), 140.7 (C-8), 50.1 (C-9), 36.1 (C-10), 21.2 (C-11), 39.6 (C-12), 42.5 (C-13), 55.9 (C-14), 23.0 (C-15), 28.9 (C-16), 56.8 (C-17), 12.0 (C-18), 19.0 (C-19), 42.2 (C-20), 21.0 (C-21), 138.3 (C-22), 129.2 (C-23), 51.2 (C-24), 31.9 (C-25), 21.1 (C-26), 19.4 (C-27), 25.4 (C-28), 12.2 (C-29) [13].

*Daucosterol* (**6**) was obtained as white powders, mp: 288–289 °C; ^1^H-NMR (400 MHz, C_5_D_5_N, ppm) δ: 5.17 (1H, m, *J* = 5.2 Hz, H-6), 4.32 (1H, d, *J* = 7.2 Hz, H-1'), 4.07 (1H, m, H-3), 0.75 (3H, d, *J* = 8.0 Hz, CH_3_-21), 0.70 (3H, s, CH_3_-19), 0.65 (6H, d, *J* = 8.4 Hz, CH_3_-26, 27), 0.59 (3H, t, *J* = 4.0Hz, CH_3_-29), 0.42 (3H, s, CH_3_-18); ^13^C-NMR (100 MHz, C_5_D_5_N, ppm) δ: 37.5 (C-1), 32.0 (C-2), 78.6 (C-3), 39.9 (C-4), 140.9 (C-5), 121.9 (C-6), 32.2 (C-7), 32.0 (C-8), 50.3 (C-9), 36.4 (C-10), 21.3 (C-11), 39.3 (C-12), 42.5 (C-13), 56.8 (C-14), 23.4 (C-15), 27.3 (C-16), 56.2 (C-17), 12.0 (C-18), 19.4 (C-19), 36.4 (C-20), 19.0 (C-21), 34.2 (C-22), 26.3 (C-23), 45.8 (C-24), 29.4 (C-25), 19.2 (C-26), 20.0 (C-27), 22.8 (C-28), 12.0 (C-29), 102.6 (C-1'), 75.3 (C-2'), 78.5 (C-3'), 71.7 (C-4'),78.0 (C-5'), 62.8 (C-6').

*Taraxerone* (**7**) was obtained as a white powder, mp: 288–289 °C; EI-MS (*m/z*)(%): 424 (M^+^, 23), 409 (M^+^-CH_3_, 17), 300 (RDA fragment, 80), 285 (70), 272 (19), 257 (20), 205 (C ring fragment, 65), 204 (100), 189 (45), 133 (62); ^1^H-NMR (500 MHz, CDCl_3_, ppm) δ: 0.82 (3H, s, CH_3_), 0.91 (3H, s, CH_3_), 0.95 (3H, s, CH_3_), 0.98 (3H, s, CH_3_), 1.08 (3H, s, CH_3_), 1.10 (3H, s, CH_3_), 1.13 (3H, s, CH_3_), 1.15 (3H, s, CH_3_), 5.56 (1H, dd, *J* = 3.2, 8.2Hz, H-15);^13^C-NMR (125 MHz, CDCl_3_, ppm) δ: 38.4 (C-1), 33.6 (C-2), 217.5 (C-3), 47.6 (C-4), 55.8 (C-5), 20.0 (C-6), 37.7 (C-7), 38.9 (C-8), 48.8 (C-9), 37.5 (C-10), 17.4 (C-11), 34.1 (C-12), 29.8 (C-13), 157.6 (C-14), 117.1 (C-15), 36.7 (C-16), 35.7 (C-17), 48.8 (C-18), 40.0 (C-19), 28.8 (C-20), 33.1 (C-21), 35.1 (C-22), 26.1 (C-23), 21.5 (C-24), 14.8 (C-25), 29.9 (C-26), 25.6 (C-27), 29.9 (C-28), 33.4 (C-29), 21.3 (C-30) [14].

### 3.4. Xanthine Oxidase Inhibitory Assay

The inhibitory effect of compound **1** against xanthine oxidase activity was carried out according to the previous investigations [15]. The experiment was performed by using 96 holes plate, and the allopurinol and DMSO were used as the positive and negative control. Firstly, xanthine (50 μmol/L) was added into each reaction system, and then the tested samples were added. After that, the xanthine oxidase (0.1 U/mL), quinoline solution (5 μmol/L), (NH_4_)_2_Fe (SO_4_) solution (1 μmol/L), H_2_SO_4_ solution (50 μmol/L) were added subsequently. The reaction system incubated at room temperature for 18 min, then added NaOH solution (1 mol/L) into total reaction system to 150 μL. Finally, adding 150 μL ethanol to terminate the reaction, and the reaction production was determined by measuring the absorbance at 380 nm by using fluorescent chemiluminescence detector on a microplate spectrophotometer. The inhibitory percentage against xanthine oxidase (%) was calculated using the following equation:

inhibition (%) = [1 − (A sample/A control) × 100

In addition, the IC_50_ values were calculated 

## 4. Conclusions

In the present study, a new cycloartane-type triterpenoid saponin named riparsaponin was isolated from the stem of *H. riparia*, and it has a significant inhibitory effect on xanthine oxidase.
